# Suitability of Habitats in Nepal for *Dactylorhiza hatagirea* Now and under Predicted Future Changes in Climate

**DOI:** 10.3390/plants10030467

**Published:** 2021-03-02

**Authors:** Bikram Shrestha, Spyros Tsiftsis, Deep Jyoti Chapagain, Chhatra Khadka, Prakash Bhattarai, Neelima Kayastha Shrestha, Marta Alicja Kolanowska, Pavel Kindlmann

**Affiliations:** 1 Department of Biodiversity Research, Global Change Research Institute CAS, Bělidla 986/4a, 603 00 Brno, Czech Republic; marta.a.kolanowska@gmail.com (M.A.K.); pavel.kindlmann@centrum.cz (P.K.); 2 Institute for Environmental Studies, Faculty of Science, Charles University, Benátská 2, 128 01 Prague, Czech Republic; 3 Department of Forest and Natural Environment Sciences, International Hellenic University, GR-66100 Drama, Greece; stsiftsis@for.ihu.gr; 4 Central Department of Botany, Tribhuvan University, Kirtipur 44618, Nepal; chapagaindeep@gmail.com (D.J.C.); light.bhattarai@gmail.com (P.B.); 5 Department of Food and Resource Economics, University of Copenhagen, 1165 Copenhagen, Denmark; 6 Chitwan National Park, Department of National Parks and Wildlife Conservation, Government of Nepal Ministry of Forests and Environment, Kathmandu 44600, Nepal; chhatra10@gmail.com; 7 Institute of Geographic Sciences and Natural Resources Research, Chinese Academy of Sciences, Beijing 100101, China; 8 Natural Product Chemistry, School of Health and Allied Sciences, Pokhara University, Pokhara 33700, Nepal; neelima.kstha@gmail.com; 9 Department of Geobotany and Plant Ecology, Faculty of Biology and Environmental Protection, University of Lodz, Banacha 12/16, 90-237 Lodz, Poland

**Keywords:** *Dactylorhiza hatagirea*, medicinal plant, Himalaya, Nepal, climate change, Orchidaceae

## Abstract

*Dactylorhiza hatagirea* is a terrestrial orchid listed in Appendix II of the Convention on International Trade in Endangered Species of Wild Fauna and Flora (CITES) and classified as threatened by International Union for Conservation of Nature (IUCN). It is endemic to the Hindu-Kush Himalayan region, distributed from Pakistan to China. The main threat to its existence is climate change and the associated change in the distribution of its suitable habitats to higher altitudes due to increasing temperature. It is therefore necessary to determine the habitats that are suitable for its survival and their expected distribution after the predicted changes in climate. To do this, we use Maxent modelling of the data for its 208 locations. We predict its distribution in 2050 and 2070 using four climate change models and two greenhouse gas concentration trajectories. This revealed severe losses of suitable habitat in Nepal, in which, under the worst scenario, there will be a 71–81% reduction the number of suitable locations for *D. hatagirea* by 2050 and 95–98% by 2070. Under the most favorable scenario, this reduction will be 65–85% by 2070. The intermediate greenhouse gas concentration trajectory surprisingly would result in a greater reduction by 2070 than the worst-case scenario. Our results provide important guidelines that local authorities interested in conserving this species could use to select areas that need to be protected now and in the future.

## 1. Introduction

The world is rapidly changing and, as a result, biodiversity is being affected in various ways [1]. In addition to habitat loss, which is characterized as the greatest threat to species [2], living creatures are also affected by the increase in human emissions of carbon dioxide due to burning fossil fuels and other greenhouse gases, which are causing climate change [1]. The potential effects of future climatic conditions on biodiversity have been widely investigated and a large number of publications exist that predict these effects on trees [3,4,5,6,7,8,9,10], medicinal herbaceous plants and shrubs [11,12,13,14,15,16,17], fungi [18], invasive plants [19,20], terrestrial orchids [21], etc.

Due to the expected increase in temperature, the suitable habitats of many species will only be available further north or at higher altitudes e.g., [19,22,23,24,25]. Those species that are not able to colonize these habitats before their present sites become unsuitable are likely to become extinct, e.g., alpine plants [11]. To assess the effects of climate change on species, the most widely used method is species distribution modelling (SDM), which links data on species distribution with environmental data and is able to predict potentially suitable areas and their changes over time [26,27]. 

It is expected that all areas in the world will be affected by climate change, but these effects will vary spatially [1]. Mountain ecosystems, particularly those at tropical latitudes, are considered ecologically sensitive and vulnerable to climate change [28]. The Himalayas are predicted to experience a rise in temperature of 5–6 °C and increase in precipitation by 20–30% by the end of the twenty-first century [29], making them one of the most threatened non-polar regions in the world [30]. Nepalese Himalayas are already being affected by rapid temperature increase and other environmental changes at both regional and local scales [30,31]. This alleviates some of the abiotic restrictions (for example, low temperature and water availability) on the activity and distributions of species [32]. Recent work has shown that the increasingly warmer climate conditions allow species to become established at higher altitudes (in the nival zone), which forms a species pool for lower altitudes [33] and may drive endemic species with restricted physiological ranges to extinction [34]. 

Members of the family Orchidaceae belong to the plant species expected to decline to a lesser or greater degree. Many of these plants, especially terrestrial orchids, depend on highly specific insect pollinators and complex mycorrhizal associations [35,36,37,38]. This makes them more vulnerable to any ecosystem degradation and increases the likelihood of their extinction. In addition, ignorance of these complex biological associations usually results in an overestimation of their potential distribution area based on SDMs [39,40,41].

*Dactylorhiza hatagirea* (D.Don) Soó is a terrestrial orchid commonly known as “Panchaunle” in Nepali. It is listed in Appendix II of the Convention on International Trade in Endangered Species of Wild Fauna and Flora (CITES). It is classified as a critically endangered species by the Conservation Assessment and Management Plan [42] and categorized as threatened by the International Union for Conservation of Nature (IUCN); [43]. Moreover, it is considered as an endangered and strictly protected species in list I of the Government of Nepal [44]. It is endemic to the Hindu-Kush Himalayan region and distributed from Pakistan to China [45]. The species occurs patchily, in very low densities of 0.1 to 2.18 individuals/m^2^ [46,47,48,49]. 

*Dactylorhiza hatagirea* grows in the pastures in the sub-alpine and alpine zones at altitudes of 2800–4200 m [50]. It is considered to be highly valuable medicinal species in Nepal. The tubers of this orchid are used for treating diabetes, dysentery, colic pain, seminal weakness, and diarrhea, and as an aphrodisiac [51,52,53]. More recent findings indicate that *D. hatagirea* also has anti-cancerous properties [54].

Due to its medicinal properties, this species has been heavily exploited. Over-exploitation, illegal trade, habitat destruction, and climate change are thus major threats to its survival [46]. *D. hatagirea* is also a good indicator of environmental quality [55]. This indicates the need to establish areas where this species will be able to survive, thus enhancing its conservation and restoration [56,57]. Therefore, information about its habitats is particularly important for ensuring its proper management and protection. 

In Nepal, only a few studies have focused on the abundance, density, and conservation of *D. hatagirea* [46,47,58], and only one on suitable habitat modelling [59]. Possible effects of climate change on its distribution have yet to be explored. Kunwar et al. [59] use bioclimatic variables and slope as predictors for identifying suitable habitats. However, they rely mostly on herbarium data, not on data collected directly in the field. In addition, they ignore geological substrate and soil properties, which are important factors determining habitat suitability for terrestrial orchids [60]. Finally, they make only an analysis of the current state and make no predictions for the future potential distribution of *D. hatagirea* under climate change.

Our study differs from Kunwar et al. [59] by:

(a) using recent data from field excursions;
(b) mapping the potential suitable habitat using more predictors than Kunwar et al. [59] by adding geological substrate and soil properties, which makes the predictions more realistic;
(c) making predictions about how the current potential distribution of this species will change under future climate change scenarios.

This is a large step forward compared to the stage set by Kunwar et al. [59]. Our results will help local authorities to effectively manage the conservation of the endangered *D. hatagirea*.

## 2. Results

### 2.1. Environmental Variables Associated with the Localities Where D. hatagirea Currently Occurs

Although the initial dataset contained a large number of localities for *D. hatagirea* (208 microsites), only 44 grid cells were occupied. This number of locations and grid cells is sufficient for almost all modelling techniques. In general, a sample size of c. 40–50 records (grids) provides robust models. Moreover, Maxent model produced good models even with smaller sample sizes [61,62]. 

The values of Pearson’s correlation coefficients between the bioclimatic variables are presented in Table 1. After omitting the variables with a high intercorrelation (r > |0.80|), those included in the Maxent software were the annual mean temperature (BIO1), the mean diurnal range (BIO2), the temperature seasonality (BIO4), the annual precipitation (BIO12), the precipitation seasonality (BIO15), the precipitation in the driest quarter (BIO17), and the precipitation in the coldest quarter (BIO19).

**Figure 1:**
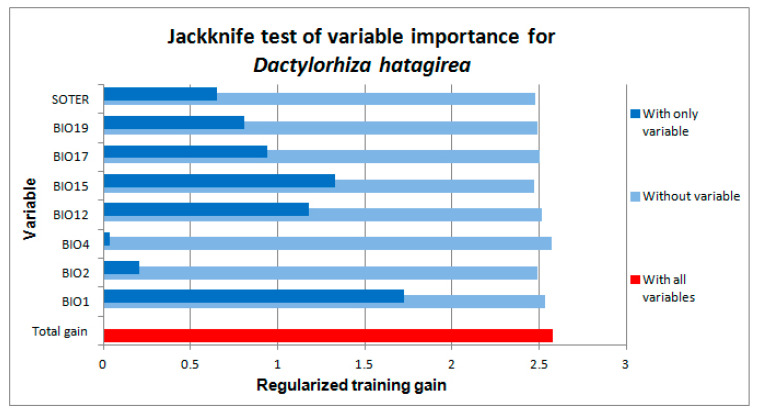
Results of the jackknife test of the importance of the environmental variables to be used in the development of the Maxent model of *Dactylorhiza hatagirea*. The length of the dark-blue bar indicates the size of the impact of the selected variable, and the length of light-blue bar shows how much of the explanatory power of the model would be lost if the corresponding factor were excluded from the analysis. See Table 1 for full names of the variables on the y-axis.

The jackknife test indicated that annual mean temperature (BIO1), precipitation seasonality (BIO15), and annual precipitation (BIO12) are the most significant variables, whereas temperature seasonality (BIO4) and the mean diurnal range (BIO2) were the least significant. The precipitation in the driest quarter (BIO17), the precipitation in the coldest quarter (BIO19), and the environmental layer of the parent material, as generated from the Soil and Terrain Database (SOTER) of Nepal, were of intermediate significance (Figure 1). Omitting precipitation seasonality (BIO15) from the model causes the largest loss in the explanatory power of the model, compared with omitting any of the remaining variables. Mean diurnal range (BIO2) and temperature seasonality (BIO4) by themselves are not useful for estimating the distribution of *D. hatagirea*. 

### 2.2. Distribution and Habitat Suitability of D. hatagirea under Current Climate and Various Future Climate Change Scenarios 

**Figure 2:**
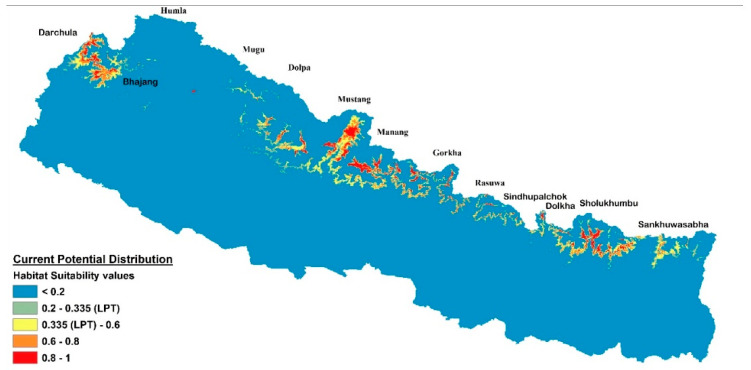
Predicted current potential distribution of habitats of different suitability in Nepal based on current climatic conditions. The values 0.2–0.335 (grey colour) indicate low suitability (below the lowest presence threshold), 0.335–0.6 (yellow colour) medium suitability, 0.6–0.8 (orange colour) high suitability, and 0.8–1 (red colour) very high suitability areas.

**Figure 3:**
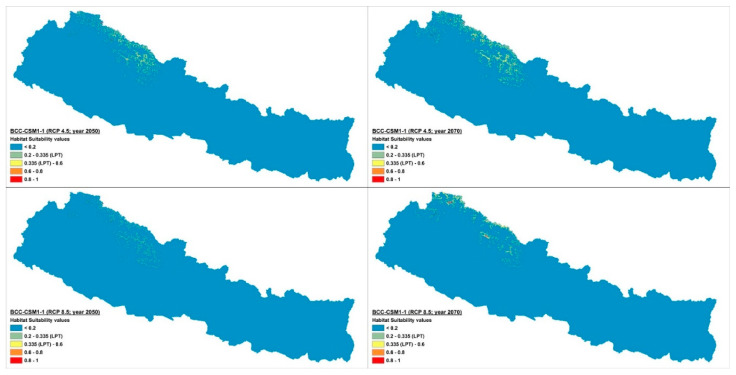
Climate change scenario under BCC-CSM1 model, RCP 4.5 and 8.5 at 2050 and 2070.

**Figure 4:**
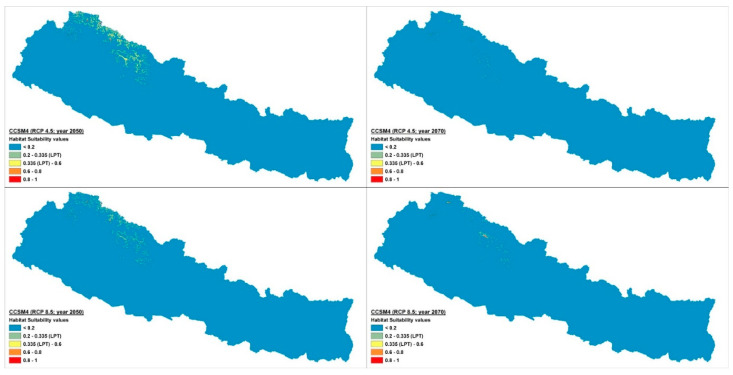
Climate change scenario under CCSM4 model, RCP 4.5 and 8.5 at 2050 and 2070.

**Figure 5:**
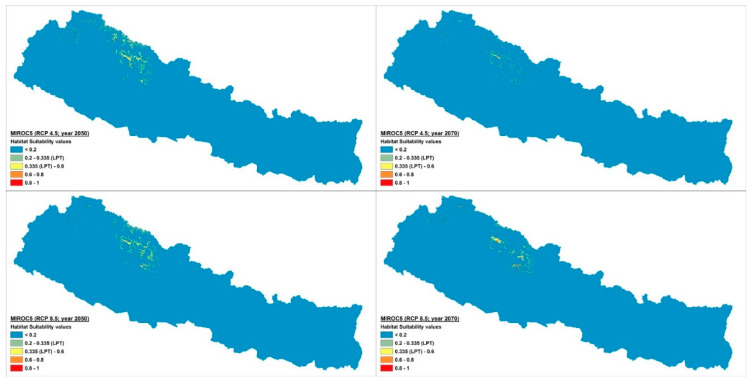
Climate change scenario under MIROC5 model, RCP 4.5 and 8.5 at 2050 and 2070.

**Figure 6:**
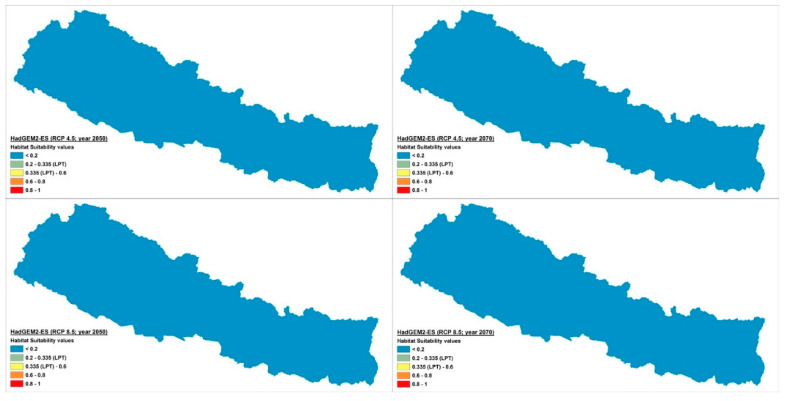
Climate change scenario under HadGEM2-ESmodel, RCP 4.5 and 8.5 at 2050 and 2070.

The current potential distribution predicted by the Maxent model is shown in Figure 2. Grid cells with habitat suitability values for *D. hatagirea* below the lowest presence threshold (LPT = 0.335) are unsuitable areas (blue and green colors). Under current climate conditions, the potentially suitable habitats are located in the districts Darchula, Bhajang, Dolpa, Magdi, Mustang, Manang, Kaski, Gorkha, Rasuwa, Sindhupalchok, Dolakha, Sholukhumbu, and Sankhuwasabha in Nepal. That is, there is an area of 10,839 km^2^ with a suitability index larger than 0.2 (Figure 2). In Kunwar et al. [59], some additional areas, covering 25,479 km^2^, are reported as being suitable: Bajung, Kalikot, Humla, Mugu, Jumla, Rukum, and Taplejung. Under future climate scenarios (Figure 3, Figure 4 and Figure 5), it is expected that suitable habitats will only remain in Dolpa region. However, the suitable areas will then include the districts Mugu and Humla (Figure 3, Figure 4 and Figure 5) and the total range of very highly suitable areas covers c. 1700 grid cells under RCP4.5 in 2070 under the BCC-CSM1-1 model (Figure 3). All of the suitable habitats (both current and new) will be collapsed in the HadGEMM2-ES model (Figure 6). 

Figures for each climate change model are shown below.

### 2.3. Numbers of Suitable Grid Cells of D. hatagirea under Current Climate vs. Those under Different Future Climate Change Scenarios

**Figure 7:**
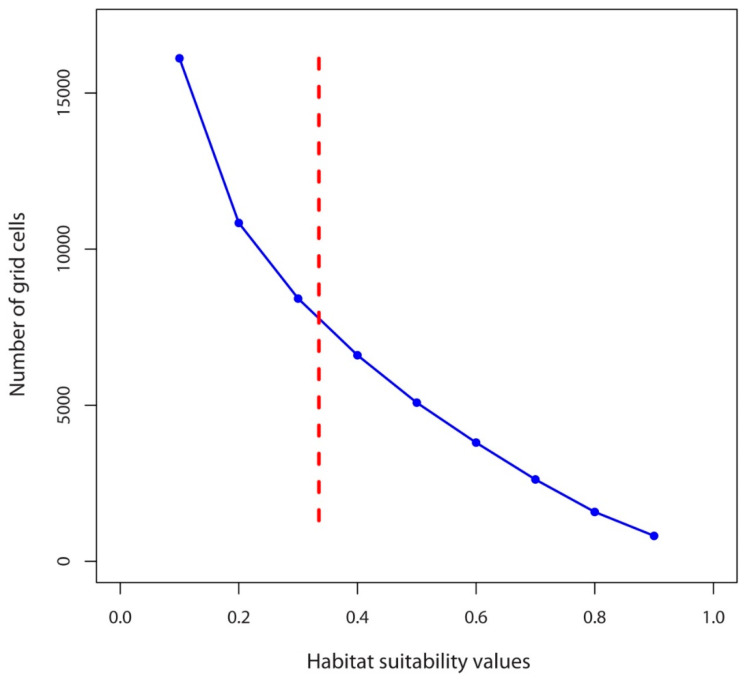
The current number of grid cells in Nepal with particular habitat suitability values. The dashed vertical red line is the lowest presence threshold (LPT = 0.335).

**Figure 8:**
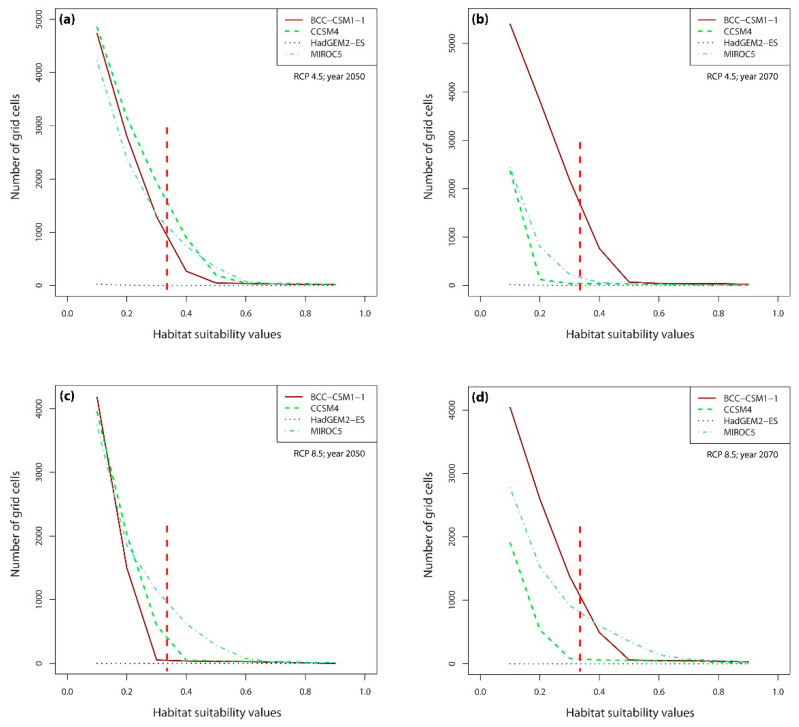
The number of grid cells in Nepal with particular habitat suitability values predicted for 2050 according to RCP4.5 (**a**) and RCP8.5 (**c**) and 2070 according to RCP4.5 (**b**) and RCP8.5 (**d**), based on four climate change models (see inset). The dashed vertical red line is the lowest presence threshold (LPT = 0.335).

Under current climatic conditions, the habitat suitability values of 7731 grid cells were above the lowest presence threshold (LPT = 0.335) (Figure 7), whereas under the future climate scenarios, the number of grid cells with potentially suitable habitats falls dramatically, reaching a maximum of ca. 1700 grid cells in 2070 under BCC-CSM1-1 (RCP4.5) scenario (Figure 3 and Figure 8b). Contrary to other climate change models, the Hadley Earth System Model (HadGEM2-ES) predicts the collapse of almost all suitable habitats under all four types of climate change scenarios (Figure 6 and Figure 8).

In RCP4.5, the numbers of grid cells containing suitable habitats ranged from 0 to 1596 in 2050 and from 0 to 1696 in 2070 in all four models of the climate change scenario considered here. In RCP8.5, this range was 0 to 949 in 2050 and 0 to 1043 in 2070. On average, there were less than 1600 grid cells containing suitable habitats in 2050 and 1700 in 2070.

### 2.4. Percentage Change in Habitat Suitability of D. hatagirea under Current Climate and under Different Future Climate Change Scenarios

**Figure 9:**
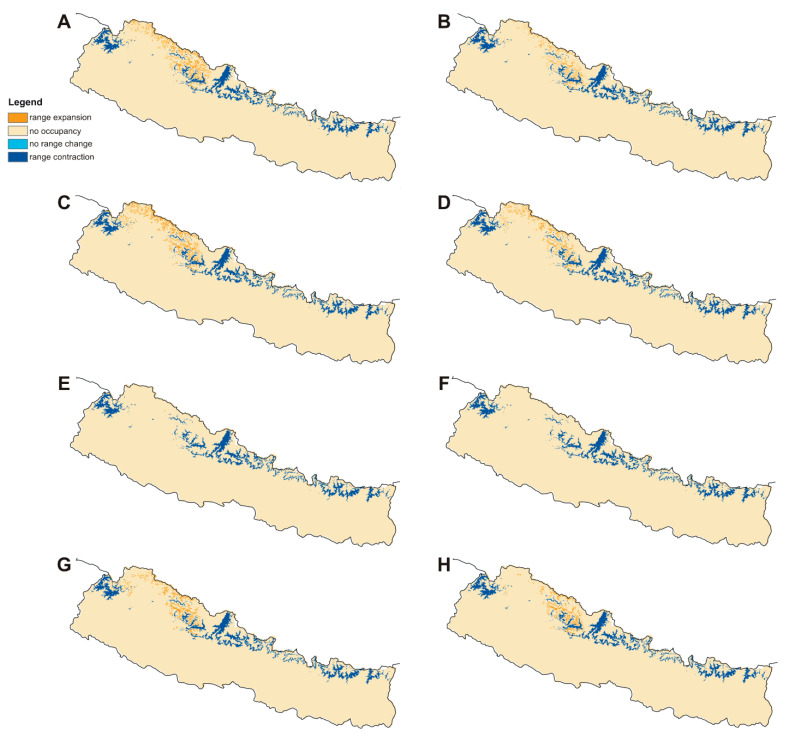
Changes in the distribution of suitable niches of *Dactylorhiza hatagirea* in 2050 according to RCP4.5 (**A**,**C**,**E**,**G**) and RCP8.5 (**B**,**D**,**F**,**H**) climate change scenarios. (**A**,**B**), BCC-CSM1-1 model; (**C**,**D**), CCSM4 model; (**E**,**F**), HadGEM2-ES model; (**G**,**H**), MIROC5 model.

**Figure 10:**
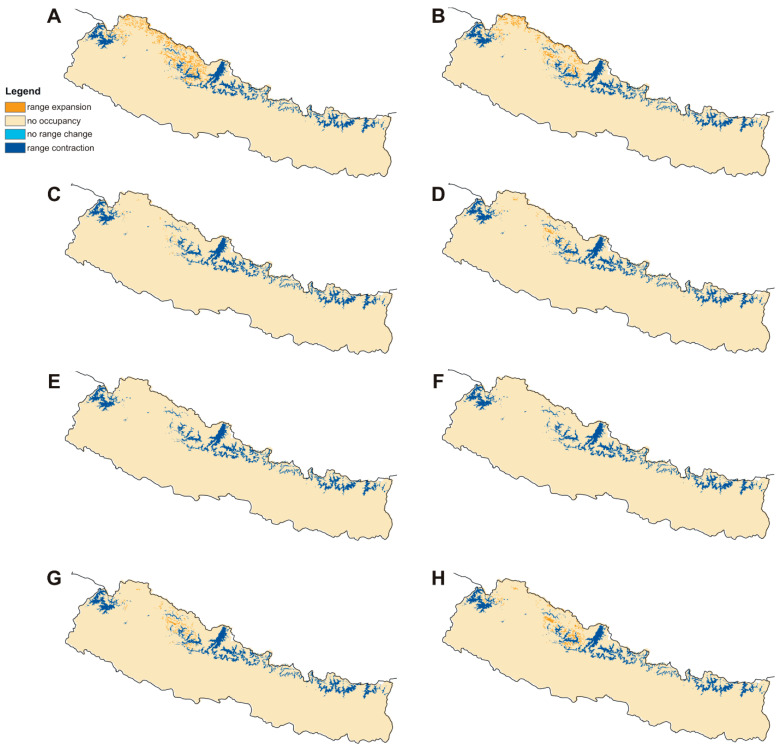
Changes in the distribution of suitable niches of *Dactylorhiza hatagirea* in 2070 according to RCP4.5 (**A**,**C**,**E**,**G**) and RCP8.5 (**B**,**D**,**F**,**H**) climate change scenarios. (**A**,**B**), BCC-CSM1-1 model; (**C**,**D**), CCSM4 model; (**E**,**F**), HadGEM2-ES model; (**G**,**H**), MIROC5 model.

Considering coverage of suitable habitats, the studied species will lose all preferred niches by 2070 in HadGEM2-ES RCP4.5 and RCP8.5 scenarios. Based on CCSM4 simulation, it will lose 71–81% of its niches by 2050 and 95–98% by 2070 (Table 2, Figure 9 and Figure 10). The most favorable for *D. hatagirea* is the BCC-CSM1-1 model. However, even in this scenario the orchid will lose 65–85% of its suitable habitats (Table 2). Surprisingly, the RCP.4.5 pathway seems to be more harmful for *D. hatagirea* occurrence in 2070 than RCP8.5. 

### 2.5. Change in Altitude of Potential Suitable Areas of D. hatagirea under Current Climate and under Different Future Climate Change Scenarios

**Figure 11:**
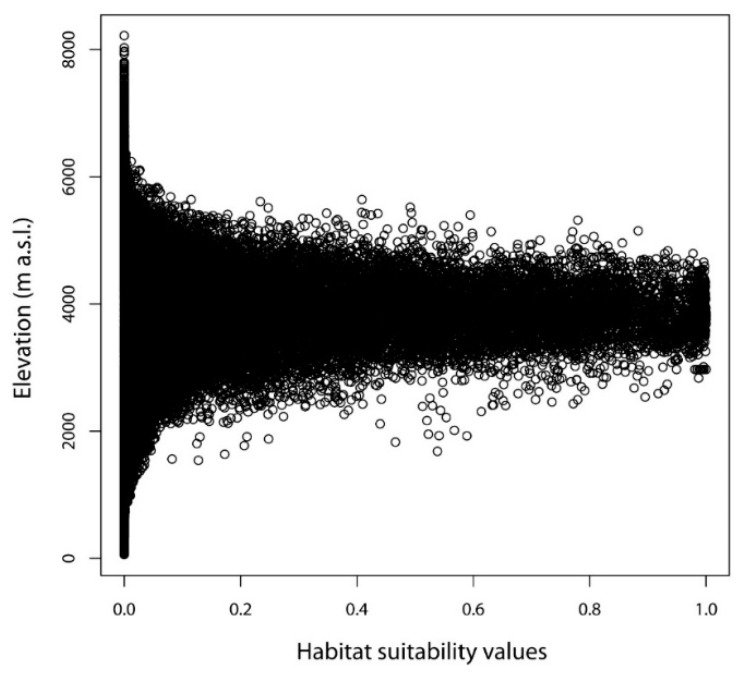
Altitude of the potential suitable areas under current climate conditions.

**Figure 12:**
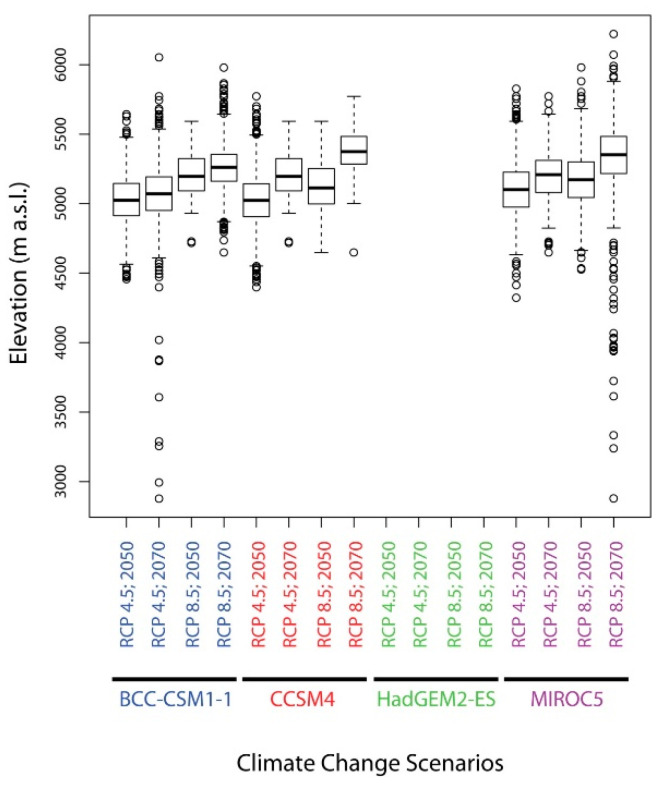
Elevational range of the grid cells where *D. hatagirea* was predicted as present (habitat suitability values ≥ 0.335) under the different climate change scenarios.

Under the current climatic conditions, the potentially suitable areas for *D. hatagirea* are located between 2300 and 5000 m. a.s.l., whereas the average altitude is at ca. 4000 m (Figure 11). Under future climate change scenarios, it is expected that *D. hatagirea* will shift towards higher altitudes, reaching up to 6000 m a.s.l., whereas the average altitude will be ca. 5000 m (Figure 12).

## 3. Discussion

The export of medicinal species from Nepal to China began in the late 1990s, and continues today [63]. Although orchids in Nepal are protected according to local legal acts (Forest Act 1993, Forest Regulations 1995, and amendment in 2001), contradicting its own policies, the Government of Nepal published a notification on 14th April 2008 permitting collection of wild orchids for trade [64]. The absence of clear regulations on sustainable harvesting and insufficient implementation of policies recently caused an increase in the illegal trade of orchids [64,65]. In addition to the direct destruction of *D. hatagirea* populations for commerce, Bhattarai et al. [46] found grazing, trampling, number of cattle, and distance to settlement as the main anthropogenic factors negatively affecting the population condition of this orchid. As indicated in our study, the survival chances of the orchid will be further reduced by climate change. Our analyses indicate severe habitat loss in Nepal and, in the worst-case scenario, all suitable niches of *D. hatagirea* will disappear. 

Current suitable habitats for *D. hatagirea* suggested by Kunwar et al. [59] are greater than ours. This is most likely because Kunwar et al. [59] used only a subset of the predicting variables used here. Although climatic factors are not the only factors determining habitat suitability [66], Kunwar et al. [59] did not consider soil properties (identified as being of intermediate significance here). 

The other result of our analyses is a predicted change in altitudinal distribution of *D. hatagirea*. Based on the environmental predictors used in the analyses (bioclimatic variables, soil type), the distribution of *D. hatagirea* was found to be mainly climate driven because the total gain of Maxent model was greatly influenced by temperature and precipitation. Thus, it is expected that *D. hatagirea* will shift towards areas of higher altitude. Specifically, the currently observed average vertical range of this species is 4000 m, whereas in the future the average altitude of the studied orchid will be elevated to 5000 m. This trend of moving uphill was already found in other plants inhabiting mountain slopes [67]. However, it is important to notice that species abundance can also change and lead to altered dynamics in the plant communities in the plant communities [68]. This might also be the case of *D. hatagirea*, which—like all orchids—is also sensitive to the increased competition. Thus, climate change may also affect *D. hatagirea* indirectly, through the change in the competition equilibrium (e.g., expansion of forest into grasslands, expansion of tall grasses). The tree-lines of the Himalaya are mainly formed by *Abies* spp., *Betula utilis, Juniperus* spp., *Pinus wallichiana, Larix* spp., *Rhododendron campanulatum*, and *Sorbus* spp. Field-based studies [69,70] have revealed an increase in tree density that is stable but with potential for shifting, in addition to upward shifting of the tree-line in the Nepalese Himalayas.

Although geological substrate and soil properties are key factors determining the distribution of several terrestrial orchids, at least at the regional and local levels, the importance of these factors differs between species [60]. For example, variation in the preferences of bedrock type was found for alpine *Dactylorhiza sambucina* [60]. This is because the occurrence of high-montane species is generally more dependent on climate, especially temperature [71]. *D. hatagirea* can occur in a number of geological substrates. Parent material (soil properties) was identified as the only factor of intermediate importance for the occurrence of *D. hatagirea* in our study and therefore cannot be ignored. 

In addition to abiotic factors, the potential distribution of *D. hatagirea* can be shaped by its interaction with other plant or animal species (e.g., plant-pollinator or/and plant-mycorrhizal fungi interactions). Bhattarai et al. [46] claim that the distribution of *D. hatagirea* is associated with the occurrence of other plant species, such as *Anemone demissa*, *Bistorta macrophylla*, *Chesneya nubigena*, *Morina nepalensis*, and *Morina polyphylla*. At present, we are not able to predict how climate change will affect these plants and how the potential changes in the local flora will disturb distribution of the studied orchid. Moreover, *D. hatagirea* is self-compatible but it is not capable of selfing [72]. The reproduction requires an animal pollen vector (probably insects, similar to other *Dactylorhiza* species), however, to date no data on specific insects transferring pollinia have been reported. Due to the lack of information on the specific composition of *D. hatagirea* pollinators, we were not able to analyze the impact of climate change on studied orchid pollen vectors, as has been done for rare European orchids [40,41]. Because pollinators are also severely threatened by climate change and habitat destruction [73], all factors that will affect their distribution or activity can be potentially considered as serious threats for *D. hatagirea*. The other question regards the influence of climate change on mycorrhizal fungi, which are crucial for seedling development in all orchids. At present, we do not know how specific are the relationships of *D. hatagirea* with symbiotic fungi and the species composition of its mycorrhizal associations. Further studies are required not only to evaluate the future distribution of orchid symbionts, but also to improve possible reintroduction of this species. 

Conservation efforts should be focused on areas that will be suitable for *D. hatagirea* occurrence in the future, as indicated in our study. Among suitable areas in 13 districts under current climate conditions, only the Dolpa region will be unchanged under future climate change. However, the new suitable areas will be extended towards Mugu and Humla districts. The local scale genetic diversity is also equally important in order to determine which areas host rare genotypes or high genetic variability, and are therefore especially worth of preservation [74]. *D. hatagirea* is a strictly protected species in Nepal and banned to local collection, trade, and processing [44]. Intensive ex situ mass propagation is likely to reduce the rhizome collection from nature and simultaneously meet the economic demand, and together with the now supported massive reintroductions might promote the survival of *D. hatagirea* [75].

In general, species distribution models identify a portion of a species’ fundamental niche, which is determined by the specific set of environmental predictors used in the analyses [76]. However, the actual distribution is always determined by complex biotic and abiotic interactions (e.g., competition, symbioses), in addition to biogeographical limitations (e.g., dispersal ability, historical factors). In our case, we used a 1 × 1 km grid. Thus, orchids may be able to survive even in areas that were identified as non-suitable in the scale we used. This might be caused by the existence of suitable micro-sites that cannot be identified when using coarser spatial resolution.

In contrast, in some cases, the outputs of species distribution models may overestimate the species distribution, which may be attributed to a poor set of predictors. Other factors that could cause such inconsistences are related to the vegetation succession. Even if vegetation types or formations are also expected to change due to climate change [69,70], predictions of these changes are very difficult and cannot be taken into consideration when we explore the effects of climate change on the distribution of a specific species. Moreover, the importance of vegetation succession is low in cases when the effects of climate change in grassland species (such as *D. hatagirea*) are considered, because forest establishment in an area is a much slower process compared to the upward shift of a grassland herbaceous plant [69,70]. Contrary to the grassland species, such changes would be very important in the case of plant species occurring within forests, as such species are expected to decline or become extinct if forests change or become smaller. 

## 4. Materials and Methods

### 4.1. Study Area

**Figure 13:**
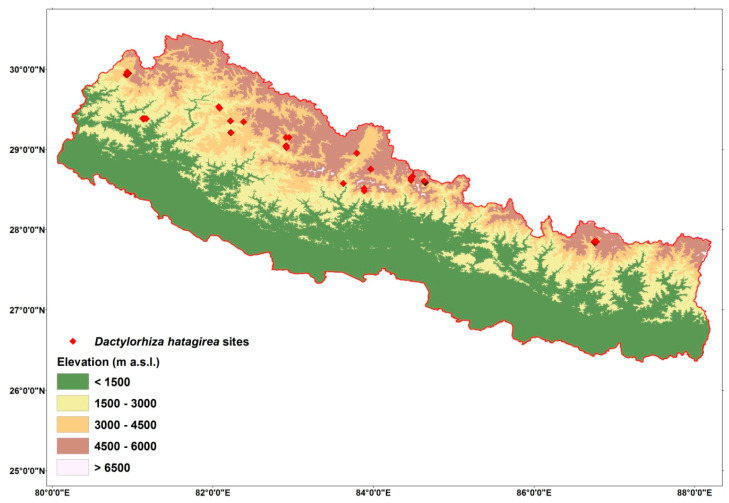
Map of the study area (Nepal) with the sites where *D. hatagirea* was found.

The study areas were in 14 locations of 3 conservation areas, 4 protected areas, and two districts which were outside of the protected areas (Figure 13). We recorded the *D. hatagirea* in Api Nampa Conservation Area (Chamelia Valley of Darchula), Khaptad National Park (Khaptad valley), Rara National Park (Rara lake), Jumla district (Depalgaun, Patamara, and Chhumchaur), Shey Phoksundo National Park (Lower Dolpa), Annapurna Conservation Area (Lower Mustang, Upper Mustang, Upper Manang, Ghandruk-Deurali), Manang district (Bhimtang), Manaslu Conservation Area (Samagaun), and Sagarmatha National Park (Phortse). All of these locations are located in sub-alpine and alpine climates of the high mountains (3000–5000 m). The major forest consists of subalpine conifer forests (<3000 m), sub-alpine mixed forest dominated by *Abies spectabilis* and *Betula utilis* (>3500–4000 m), and alpine shrub and meadows dominated by dwarf rhododendrons and alpine herbs and grass (3000–5000 m).

### 4.2. Sampling and GPS Presence Locations

*Dactylorhiza hatagirea* is a well-known medicinal plant which is commonly collected by local people [51,52,53]. Based on information obtained from collectors of this medicinal herb, we travelled along main trails used by people and herder trails in pasturelands in the 14 locations of the studied area with altitudinal range between 2200 and 5500 m to locate the presence of *Dactylorhiza hatagirea*. Once we recorded the presence areas, all presence GPS locations were recorded using a handheld device GARMIN GPSMAP. Moreover, we used distribution data of our research team obtained during field excursions conducted over the past 15 years. The main aim of our sampling was to record *D. hatagirea* in as many areas as possible. This is because our modelling analysis (described below) is based on ecological information obtained from a number of different sites and these data are used to predict the potential distribution of a species by calculating the habitat suitability across the landscape. 

### 4.3. Predictive Distribution Model

To explore the potentially suitable area for *Dactylorhiza hatagirea* in Nepal under current and future climatic conditions, we applied ecological niche models generated by Maxent software version 3.4.1 [77,78].

Previous studies have used a variety of techniques and statistical methods have been used for the species distribution modelling [79,80,81,82,83]. They are based on resource selection function [84,85], generalized linear models [86], algorithmic modelling based on machine learning [87], and a maximum entropy model called Maxent [78]. Among these, currently the most often used method is Maxent because of its efficiency to handle complex interactions between response and predictor variables, and possibility to be integrated with GIS techniques [11]. 

Maxent is used in modelling species distributions and is characterized by a high predictive performance, even in the case of very small sample sizes [61,62,88,89]. The model uses presence-only occurrence data and a set of environmental variables, and by using the maximum entropy principle it computes habitat suitability values over the entire study area.

The distribution data used for the model were based on the sampling mentioned above. As the first step, we used 20 environmental explanatory variables. Nineteen of these were bioclimatic variables and one corresponded to the soil properties of the study area. The bioclimatic variables were downloaded from the WorldClim database [90] in a 30-sec resolution (approximately 1 km^2^), whereas the environmental layer corresponding to the soil properties was derived from the soil and terrain database (SOTER) for Nepal at a scale of 1:1,000,000 [91]. Although SOTER for Nepal was in vector format, the layer containing information about the parent material was converted to raster format at the same resolution and extent with the layers of the bioclimatic variables. Because specific bioclimatic variables are usually highly intercorrelated with others, we used ENMTools package [92] to calculate Pearson correlation coefficients for all pairwise interactions. To eliminate highly correlated variables, only one (i.e., the one with the higher percent contribution and training gain) was selected among any pair of those with a correlation coefficient r > |0.80| [5,9].

In total, ten models were run with Maxent, and as suggested by Phillips and Dudík [71], we used the auto-features mode and the default settings. Maxent models were run using 80% of the sample point data and tested against the remainder. Because of the rather small number of occurrences of *D. hatagirea*, bootstrapping was used as a form of replication (test samples chosen randomly by sampling with replacement). We assessed model performance in the ENMTools package [92] using the Akaike information criterion (AICc) because it greatly outperforms BIC and AUC based methods, especially when sample size is small [93]. 

The future potential distribution of *D. hatagirea* and its habitat suitability in Nepal was predicted using 16 climate change scenarios, corresponding to the years 2050 (average for 2041–2060) and 2070 (average for 2061–2080). Specifically, for each period (2050 and 2070), four climate change models were chosen under the fifth Coupled Model Intercomparison Project (CMIP5). We chose the following climate change models: (a) the Hadley Earth System Model (HadGEM2-ES), (b) the Community Change System Model (CCSM4), (c) the Beijing Climate Centre Climate System Model (BCC-CSM1-1), and (d) the Model for Interdisciplinary Research on Climate (MIROC5). Two Representative Concentration Pathways (RCPs) were used for each period and each climate change model. The four different pathways that are available differ in the amounts of greenhouse gases that are expected to be emitted in the future. Beginning from the RCP2.6 to the RCP8.5, the emissions increase and thus RCP2.6 is a low emission scenario, whereas the RCP8.5 is a maximum emission scenario. The other two emission scenarios (RCP4.5 and RCP6.0) fall between these two extremes. In our analyses, we used the maximum greenhouse gas emission scenario (RCP8.5) and one out of the two medium greenhouse gas emission scenarios. Following Jiang et al. [94], and their results for China, the RCP4.5 was preferred instead of the other medium gas emission scenario (RCP6.0).

The outcome of the species distribution models is a continuous suitability map, which is usually converted into presence/absence data [76]. In the present study, the lowest presence threshold (equaling the minimum training presence) was used because it provides a reliable distribution of the studied species and can be better interpreted ecologically [95].

SDMtoolbox 2.3 for ArcGIS [96] was used to visualize changes in the distribution of suitable niches of the studied orchid and its pollinators caused by climate change in RCP4.5 and RCP8.5 climate change scenarios. To compare distribution models created for current climatic conditions with future models, all SDMs were converted into binary rasters and projected using the Goode homolosine projection. The presence threshold was set as equal to the minimum training presence.

## 5. Conclusions

We evaluated the effect of predicted climate changes on the distribution of an endangered plant, *D. hatagirea*, in Nepal, which is already seriously threatened by illegal harvesting. The annual mean temperature (BIO1), seasonal nature of the precipitation (BIO15), and annual precipitation (BIO12) were identified as the most significant variables affecting the distribution of *D. hatagirea*. As indicated by our analyses, climate change constitutes a severe threat for this orchid. Our research predicts severe habitat loss in Nepal and, in the worst-case scenario, there are no suitable habitats for *D. hatagirea*. The coverage of suitable habitat will be zero by 2070 based on the HadGEM2-ES RCP4.5 and RCP8.5 scenarios. Based on the CCSM4 simulation, the decrease will be 71–81% by 2050 and 95–98% by 2070. The most favorable scenario is that predicted by the BCC-CSM1-1 model, which predicts a reduction of 65–85%. Surprisingly, the prediction of the occurrence of *D. hatagirea* based on the RCP.4.5 is lower in 2070 than that based on RCP8.5. 

Other factors that could affect the future distribution of *D. hatagirea* are ecological interactions between this and other species, especially specific pollinators and mycorrhizal fungi. Alterations in the distributions of the orchid’s insect pollinators could decrease its reproductive success and climate change may adversely affect its association with mycorrhizal fungi, resulting in poor seedling development.

In our opinion, conservation should focus on areas that this study indicates will be suitable for *D. hatagirea* in the future.

## Figures and Tables

**Table 1:** Pearson’s correlation coefficients among the 19 bioclimatic variables (correlations with r ≥ |0.80| are shown in bold).

	Bio1	Bio2	Bio3	Bio4	Bio5	Bio6	Bio7	Bio8	Bio9	Bio10	Bio11	Bio12	Bio13	Bio14	Bio15	Bio16	Bio17	Bio18	Bio19
Bio1	0	−0.04	0.62	−0.69	**0.99**	**0.99**	−0.58	**0.98**	**0.96**	**0.99**	**0.99**	0.78	**0.84**	−0.16	0.78	**0.83**	−0.06	0.69	−0.66
Bio2	0	0	0.19	0.32	0.05	−0.13	0.54	−0.03	−0.04	0.003	−0.08	−0.18	−0.10	−0.33	0.01	−0.11	−0.15	−0.24	−0.03
Bio3	0	0	0	**−0.81**	0.56	0.67	−0.68	0.55	0.49	0.56	0.67	0.55	0.51	0.10	0.32	0.54	0.15	0.51	−0.47
Bio4	0	0	0	0	−0.59	−0.78	**0.95**	−0.61	−0.57	−0.61	−0.75	−0.65	−0.57	−0.25	−0.31	−0.60	−0.20	−0.63	0.49
Bio5	0	0	0	0	0	**0.96**	−0.46	**0.97**	**0.96**	**0.99**	**0.97**	0.77	**0.82**	−0.23	**0.81**	**0.81**	−0.11	0.66	−0.66
Bio6	0	0	0	0	0	0	−0.69	**0.96**	**0.93**	**0.97**	**0.99**	**0.82**	**0.83**	−0.08	0.73	**0.82**	−0.01	0.72	−0.64
Bio7	0	0	0	0	0	0	0	−0.51	−0.47	−0.50	−0.65	−0.60	−0.51	−0.33	−0.25	−0.54	−0.26	−0.60	0.36
Bio8	0	0	0	0	0	0	0	0	**0.95**	**0.98**	**0.96**	0.79	**0.83**	−0.18	**0.81**	**0.82**	−0.09	0.69	−0.65
Bio9	0	0	0	0	0	0	0	0	0	**0.97**	**0.94**	0.76	**0.81**	−0.22	0.78	0.79	−0.13	0.65	−0.61
Bio10	0	0	0	0	0	0	0	0	0	0	**0.98**	0.79	**0.83**	−0.21	**0.81**	**0.82**	−0.09	0.67	−0.65
Bio11	0	0	0	0	0	0	0	0	0	0	0	**0.81**	**0.83**	−0.11	0.74	**0.82**	−0.03	0.71	−0.66
Bio12	0	0	0	0	0	0	0	0	0	0	0	0	**0.97**	−0.03	0.74	**0.98**	−0.10	**0.95**	−0.61
Bio13	0	0	0	0	0	0	0	0	0	0	0	0	0	−0.14	**0.84**	**0.99**	−0.18	**0.91**	−0.64
Bio14	0	0	0	0	0	0	0	0	0	0	0	0	0	0	−0.41	−0.13	**0.81**	−0.03	0.54
Bio15	0	0	0	0	0	0	0	0	0	0	0	0	0	0	0	**0.82**	−0.38	0.67	−0.67
Bio16	0	0	0	0	0	0	0	0	0	0	0	0	0	0	0	0	−0.18	**0.94**	−0.66
Bio17	0	0	0	0	0	0	0	0	0	0	0	0	0	0	0	0	0	−0.19	0.67
Bio18	0	0	0	0	0	0	0	0	0	0	0	0	0	0	0	0	0	0	−0.63
Bio19	0	0	0	0	0	0	0	0	0	0	0	0	0	0	0	0	0	0	0

Abbreviations: Bio1 = annual mean temperature; Bio2 = mean diurnal range; Bio3 = isothermality; Bio4 = temperature seasonality; Bio5 = maximum temperature in the warmest month; Bio6 = minimum temperature in the coldest month; Bio7 = temperature annual range; Bio8 = mean temperature in the wettest quarter; Bio9 = mean temperature in the driest quarter; Bio10 = mean temperature in the warmest quarter; Bio11 = mean temperature in the coldest quarter; Bio12 = annual precipitation; Bio13 = precipitation of the wettest month; Bio14 = precipitation of the driest month; Bio15 = precipitation seasonality; Bio16 = precipitation of the wettest quarter; Bio17 = precipitation of the driest quarter; Bio18 =precipitation of the warmest quarter; Bio19 = precipitation of the coldest quarter.

**Table 2:** Change in the coverage (km^2^) of suitable niches of *D. hatagirea* in response to various climate change scenarios.

Model	Year	RCP Pathway	−1 (Range Expansion)	0 (Absence in Both)	1 (Present in Both)	2 (Range Contraction)	Coverage Change
BCC-CSM1-1	2050	4.5	2,085,974	136,487,198	3805	8,143,194	−74.35%
		8.5	1,133,826	137,439,346	12,366	8,134,633	−85.93%
	2070	4.5	2,818,395	135,754,777	15,219	8,131,780	−65.22%
		8.5	1,890,028	136,683,145	12,366	8,134,633	−76.65%
CCSM4	2050	4.5	2,330,432	136,242,741	3805	8,143,194	−71.35%
		8.5	1,502,891	137,070,282	1902	8,145,096	−81.53%
	2070	4.5	91,315	138,481,858	0951	8,146,048	−98.87%
		8.5	389,991	138,183,182	5707	8,141,292	−95.14%
HadGEM2-ES	2050	4.5	4756	138,568,416	0	8,146,999	−99.94%
		8.5	0	138,573,172	0	8,146,999	−100.00%
	2070	4.5	0	138,573,172	0	8,146,999	−100.00%
		8.5	0	138,573,172	0	8,146,999	−100.00%
MIROC5	2050	4.5	1,776,835	136,796,337	3805	8,143,194	−78.14%
		8.5	1,385,893	137,187,279	8561	8,138,438	−82.88%
	2070	4.5	605,912	137,967,260	3805	8,143,194	−92.52%
		8.5	1,122,412	137,450,760	16,170	8,130,828	−86.02%

## Data Availability

The occurrence data of *D. hatagirea* presented in this study are available on request from the corresponding author.
